# Safety Overview of a Recombinant Live-Attenuated Tetravalent Dengue Vaccine: Pooled Analysis of Data from 18 Clinical Trials

**DOI:** 10.1371/journal.pntd.0004821

**Published:** 2016-07-14

**Authors:** Sophia Gailhardou, Anna Skipetrova, Gustavo H. Dayan, John Jezorwski, Melanie Saville, Diane Van der Vliet, T. Anh Wartel

**Affiliations:** 1 Sanofi Pasteur, Lyon, France; 2 Sanofi Pasteur, Swiftwater, Pennsylvania, United States of America; 3 Sanofi Pasteur, Marcy l’Etoile, France; 4 Sanofi Pasteur, Singapore; University of California, Davis, UNITED STATES

## Abstract

A recombinant live attenuated tetravalent dengue vaccine (CYD-TDV) has been shown to be efficacious in preventing virologically-confirmed dengue disease, severe dengue disease and dengue hospitalization in children aged 2–16 years in Asia and Latin America. We analyzed pooled safety data from 18 phase I, II and III clinical trials in which the dengue vaccine was administered to participants aged 2–60 years, including long-term safety follow-up in three efficacy trials. The participants were analyzed according to their age at enrollment. The percentage of participants aged 2–60 years reporting ≥1 solicited injection-site or systemic reactions was slightly higher in the CYD-TDV group than in the placebo group. The most common solicited injection-site reactions were pain. Headache and malaise were the most common solicited systemic reactions. In both groups 0.3% of participants discontinued for safety reasons. The most common unsolicited adverse events were injection-site reactions, gastrointestinal disorders, and infections. Reactogenicity did not increase with successive doses of CYD-TDV. The frequency and nature of SAEs occurring within 28 days of any dose were similar in the CYD-TDV and placebo groups and were common medical conditions that could be expected as a function of age. Baseline dengue virus serostatus did not appear to influence the safety profile. No vaccine-related anaphylactic reactions, neurotropic events or viscerotropic events were reported. In year 3 after dose 1, an imbalance for dengue hospitalization, including for severe dengue, observed in participants aged <9 years in the CYD-TDV group compared with the placebo group was not observed for participants aged ≥9 years. In Year 4, this imbalance in participants aged <9 years was less marked, giving an overall lower risk of dengue hospitalization or severe dengue from dose 1 to Year 4 in the CYD-TDV group. These results have contributed to the definition of the target population for vaccination (≥9 years old) for which CYD-TDV has a satisfactory safety profile. Long-term safety will continue to be monitored in the ongoing follow-up of efficacy trials. Safety and effectiveness in real-life settings will be assessed through post-licensure studies.

## Author Summary

Dengue is a mosquito-borne viral infection in tropical and subtropical regions of the world that causes flu-like illness and, in severe cases, death. Every year, 390 million dengue infections occur worldwide, and over 3.9 billion people are now at risk from the disease. Although mosquito control and improved care have helped reduce dengue’s impact, a vaccine is needed. CYD-TDV, a live-attenuated vaccine, has been shown to be efficacious in preventing symptomatic virologically-confirmed dengue disease in children aged 2–16 years. To help establish its safety, we analyzed integrated data from available clinical trials, with emphasis on safety parameters suggested by the World Health Organization. The analysis included data for 77,234 doses in 26,356 participants aged 2–60 years who have received ≥1 dose. We found that the overall safety profile of the dengue vaccine is satisfactory. The imbalance observed for dengue hospitalization and severe dengue observed in year 3 after the first dose in children aged <9 years who had received CYD-TDV vaccine was less marked in year 4. This imbalance was not observed in participants aged ≥9 years in either year. These results have contributed to the definition of the target population for vaccination, i.e. ≥9 years old for which the CYD-TDV vaccine has a satisfactory safety profile.

## Introduction

Dengue is a viral infection found in tropical and subtropical regions of the world that causes flu-like illness and can, in severe cases, result in death [1]. The disease is caused by four serotypes of dengue virus (*Flaviviridae*), DENV1-4 which are transmitted by *Aedes* mosquitoes. The incidence of dengue has grown dramatically over the last few decades. Results from a recent study suggest that 390 million dengue infections occur worldwide each year of which 96 million have clinical manifestations [2]. Another study estimated that about 3.9 billion people are now at risk [3]. Approximately 500,000 people, mostly children, are hospitalized with severe dengue, and approximately 2.5% of them die [4]. Mosquito control and improved clinical management have helped reduce the burden of dengue, but it remains a major public health concern in Asia and the Americas [4].

Among the candidate dengue vaccines, a recombinant, live-attenuated tetravalent dengue vaccine (CYD-TDV) has been tested in several phase I, II, and III clinical trials since 2002 [5–17]. This candidate vaccine is composed of four recombinant vaccine viruses (DENV1–4), each of which expresses the pre-membrane and envelope genes of a single serotype, with a yellow fever virus (YFV) 17D backbone. Pre-clinical studies have demonstrated that CYD-TDV is genetically and phenotypically stable, non-hepatotropic, and less neurovirulent than the parental 17D YFV [18].

The efficacy and safety, up to 25-months after the first dose of a three-dose vaccination regimen of CYD-TDV at D0, M6 and M12 was assessed in a monocenter phase IIb trial in Asia and two large-scale pivotal phase III efficacy trials in Asia and Latin America [19–21]. In addition, preliminary results from on-going longer-term assessment of vaccine safety in these trials, including an extension trial of the phase IIb, have been reported [22].

The World Health Organization (WHO) states that specific potential safety issues should be taken into considerations in the assessment of candidate dengue vaccines and long-term safety surveillance [23, 24]. These issues include possible vaccine-associated dengue-like disease due to vaccine viremia, sensitization or enhancement of dengue illness, and an increased likelihood of severe disease in young children. In addition, for the CYD-TDV vaccine, viscerotropic and neurotropic disease should be assessed due to the YFV backbone of the CYD-TDV. Here we report a pooled analysis of safety data from 18 phase I, II and III clinical trials that assessed the candidate CYD-TDV vaccine. The specific purpose of this pooled analysis was to increase the power to detect potential safety signals and provide more precise estimates of adverse event (AE) rates than those available in individual trials.

## Materials and Methods

### Trials included in the analysis

A total of 18 clinical trials were included in this pooled safety analysis (Table 1). In addition, the participants from a phase IIb, proof of concept, efficacy trial in Thailand, and the two phase III large-scale efficacy trials in Asia and Latin America were invited to participate in a long-term follow-up period [22]. During this long-term follow-up the participants did not receive any further vaccine or placebo injections and they were analyzed according to their original group (CYD-TDV or placebo) and their age enrollment.

**Table 1:** Summary of the characteristics of clinical trials included in the analysis.

Clinical trials; Registry ID and study code [ref]	Trial designs	Aims	Acute febrile illness case definition	Country	N	Age range (years)	Planned duration of follow-up–Data availability
Phase I trials							
CYD04 [11]	Monocentric, placebo-controlled, observer-blind (1^st^ injection), open (2^nd^ and 3^rd^ injections)	Safety, vaccine viremia, viral shedding, immunogenicity	None	USA	66	18–45	28-days after vaccination–Final data available
Eudra-CT: 2014-001534-29 CYD05 [6]	Monocentric, active vaccine (typhoid)-controlled, observer-blind (1^st^ injection), open (2^nd^ and 3^rd^ injections)	Safety, vaccine viremia and immunogenicity after every injection; 5-years post-dose 3: antibody persistence and safety; From year 1 to 4 years post-dose 3: symptomatic dengue. From 4-years to 5-years post-dose 3: hospitalized dengue cases	Temperature ≥38°C for ≥48 hours	Philippines	126	2–45	5-years post-dose 3 –Final data available
Eudra-CT: 2014-001706-17 CYD06 [25]	Monocentric, controlled, observer-blind (1^st^ injection: CYD-TDV or YF), open (2^nd^ and 3^rd^ injections: CYD-TDV for all)	Safety, vaccine viremia and immunogenicity after every injection	None	Mexico	126	2–45	28-days post-dose 3 –Final data available
Phase II trials							
NCT00730288 CYD10 [13]	Phase IIa, monocentric, open trial	Safety, vaccine viremia and immunogenicity after one injection, 6-month post-injection: safety	None	Australia	35	18–40	6-months post-dose 3 –Final data available
NCT00740155 CYD11 [26]	Phase IIa, monocentric, randomized open trial	Safety, vaccine viremia and immunogenicity after each injection: safety	None	Mexico	155	18–45	8.5-months post-dose 1 –Final data available
NCT00617344 CYD12* [8]	Randomized, not controlled.	Formulation finding	None	USA	260	18–45	6-months post-dose 3 –Final data available
NCT00993447 CYD13* [16]	Randomized, placebo and active controlled, observer-blind (dose 1 & 2), single blind (dose 3) multicenter trial	Safety and immunogenicity after each injection; Symptomatic dengue	Temperature ≥38°C lasting ≥2 days with a suspicion of dengue without evidence of local infection	Columbia, Honduras, Mexico, Puerto Rico	600	9–16	6-months post-dose 3–Final data available
NCT00875524 CYD22* [15]	Randomized, active and placebo controlled observer-blind	Safety and immunogenicity after each injection: antibody persistence and safety; Symptomatic dengue	Temperature ≥38°C for ≥48 hours with a suspicion of dengue	Vietnam	180	2–45	4-years post-dose 3 –Final data available
NCT00788151 CYD24* [9]	Randomized, active and placebo controlled, observer blind, monocenter trial	Immunogenicity after each injection, in children previously vaccinated with YF; Safety after each injection; Vaccine viremia, after dose 1 & 2, (subset); Symptomatic dengue	Temperature ≥38°C for ≥48 hours	Peru	300	2–11	6 months post-dose 3 –Final data available
NCT00880893 CYD28* [10, 27]	Randomized, active and placebo controlled, observer-blind (dose 1); single-blind (doses 2 & 3), multicenter trial	Safety, immunogenicity (subset) after each dose; immunogenicity (subset) and safety; Symptomatic, hospitalized dengue	Acute febrile illness (temperature ≥38°C) on ≥2 consecutive days, without evidence of local infection and with sign(s) of severity requiring hospitalization (with bed attribution)	Singapore	1198	2–45	4-years post-dose 3 –Final data available
NCT01187433 CYD30* [7]	Randomized, placebo-controlled, observer-blind monocenter trial	Safety and immunogenicity after each dose; Safety; Symptomatic dengue	Temperature ≥38°C on ≥2 consecutive days with a suspicion of dengue	Brazil	150	9–16	6-months post-dose 3–Final data available
NCT01550289 CYD47* [28]	Randomized, placebo-controlled, observer-blind multicenter trial	Safety and immunogenicity after each dose; Safety; Symptomatic dengue	Temperature ≥38°C on ≥2 consecutive days	India	189	18–45	6-months post-dose 3–Final data available
NCT01488890 CYD51* (see S1 Table)	Randomized, open-label, multicenter trial	Immunity after dose 3 in YF vaccinated and unvaccinated subjects; antibody persistence, in YF vaccinated and unvaccinated subjects; YF immune response at baseline and 28 days after each dose in YF+ subjects with D0, M6, M12 or D0, M2, M6 CYD-TDV schedules; YF immune response at baseline and 1, 3, and 7 months after YF vaccine at D0 with and without CYD-TDV (D0, M2, M6); Safety profile after each dose	None	USA	390	18–45	6-months post-dose 3 –Final data available
Phase IIb							
NCT00842530 CYD23* [21]	Randomized, active and placebo controlled, observer-blind monocenter trial	Efficacy proof-of-concept (symptomatic, virologically-confirmed dengue); Immunogenicity, reactogenicity (after all doses) and vaccine viremia (after dose 1 & 2) in a subset; Safety	Acute febrile illness with fever lasting for ≥1 day (temperature ≥ 37.5°C measured at ≥2 times with an interval of ≥4 hours)	Thailand	4002	4–11	13-months post-dose 3 –Final data available
NCT01983553 CYD57 [22]	Safety follow-up of CYD23	Safety, hospitalized dengue, vaccine-related and serious AEs	Acute febrile illness with fever lasting for ≥1 day (temperature ≥ 37.5°C measured at ≥2 times with an interval of ≥4 hours) requiring hospitalization	Thailand	3203	4–11 (at enrollment)	5-years post-dose 3 in CYD23 –Data for up to 4-years post-dose 1 available
Phase III trials							
NCT01254422 CYD32* [29]	Randomized, placebo-controlled, observer-blind, multicenter trial	Safety; Immunogenicity post-dose 2 & 3; Symptomatic dengue	6-months post-dose 3: dengue disease reported as SAE; 13-months post-dose 3: acute febrile illness (i.e., ≥least 2 consecutive days)	Malaysia	250	2–11	6-months post-dose–Final data available
NCT01134263 CYD17* [30]	Randomized, placebo-controlled, observer-blind, multicenter trial	Lot consistency (and bridging between phase II and III lots); Safety; Vaccine viremia and virus shedding (subset); Immunogenicity after dose 3 by baseline flavivirus immune status (subset)	None	Australia	715	18–60	6-months post-dose 3–Final data available
NCT01373281 CYD14* [19, 22]	Randomized, placebo-controlled, observer-blind, multicenter trial	Vaccine efficacy: virologically-confirmed dengue; Safety throughout the trial; Reactogenicity (injection site and systemic) after each dose (subset); Immunity after dose 2 & 3 (subset); 5-year post-dose 3: safety, confirmed-hospitalized dengue (all); antibody persistence (subset)	Up to 25 months post-dose1: Acute febrile illness (temperature ≥38°C on ≥2 consecutive days); Long-term follow-up (12-months post-dose 3 onwards): as above and requiring hospitalization	Indonesia, Malaysia, Thailand, the Philippines, Viet Nam	10,275	2–11	5-years post-dose 3 –Data for up to year 4 post-dose 1 available
NCT01374516 CYD15* [20, 22]	Randomized, placebo-controlled, observer-blind, multicenter trial	Vaccine efficacy: virologically-confirmed dengue; Safety throughout the trial; Reactogenicity (injection site and systemic) after each dose (subset); Immunity after dose 2 & 3 (subset); 5-year post-dose 3: safety, confirmed-hospitalized dengue (all); antibody persistence (subset)	Up to 25 months post-dose1: Acute febrile illness (temperature ≥38°C on ≥2 consecutive days); Long-term follow-up (12-months post-dose 3 onwards): as above and requiring hospitalization	Brazil, Colombia, Honduras, Mexico, Puerto Rico	20,689	9–16	5-years post-dose 3 –Data for up to year 3 post-dose 1 available

* Main trials, that assessed the current formulation of the CYD-TDV vaccine (containing about 5 log_10_ CCID_50_ of each of the four live, attenuated vaccine virus) administered at D0, M6 and M12; in the other trials (secondary trials) the same formulation was assessed but with administration at D0, M3.5/4 and M12.

#### Main clinical trials

The thirteen main randomized trials assessed the CYD-TDV vaccine with the current formulation (about 5 log10 CCID50 per dose of each serotype) and the current three-dose schedule, i.e. D0, M6 and M12 (Table 1).

#### Secondary clinical trials

The five secondary trials assessed the same formulation; three randomized trials with a 3-dose schedule in which the second dose was given at 3.5 or 4 months, one non-randomized trial with two doses at D0 and M3.5 and one randomized trial with one dose of CYD-TDV at D0 (Table 1).

#### Longer-term follow-up data

Long-term follow-up data are available from one phase I (CYD05; 5-years post-dose 1) and two phase II trials (CYD22 and CYD28; 4-years post-dose 1) for hospitalization for dengue disease, identified using a passive surveillance system (Table 1). Long-term data for dengue hospitalization and severe dengue are being collected, using an active surveillance system, in two of the phase III trials (CYD14 and CYD15) and the follow-up study for the phase IIb trial (CYD23/57; Table 1). Participants are contacted at least once every three months and they attend an annual visit [22]. Data are available for up to year 3 post-dose 1 in CYD15, and for up to year 4 post-dose 1 in CYD23/57 and CYD14. Due to the differences in the surveillance systems, only data from the efficacy trials were pooled in the present analysis; the number of events occurring in the other trials was provided.

#### Trial characteristics

In the phase IIb and two of the phase III efficacy trials, participants were randomized 2:1 to the CYD-TDV and placebo groups, respectively [19–21]. The trials with a control group were observer-blinded so that participants, parents, investigators, and other personnel involved in follow-up and safety evaluation were unaware of which treatment was administered to minimize bias (Table 1). One trial was non-randomized, with participants analyzed on the basis of previous vaccination with a monovalent DENV1 or DENV2 vaccine, YFV vaccine or flavivirus sero-negative status [13].

All trials except one were registered on ClinicalTrials.gov or EudraCT [11]. All 18 trials involving vaccination included in this analysis were approved by the relevant ethical review committees, institutional review boards, or both, as well as by national health autorities. They were carried out in accordance with the relevant International Conference for Harmonization guidelines and the principles of the Declaration of Helsinksi.

### CYD-TDV vaccine

The CYD-TDV vaccine candidate contained about 5 log_10_ CCID_50_ of each live, attenuated dengue vaccine virus (serotypes 1–4) [5, 18]. The vaccine was supplied as a freeze-dried powder and was reconstituted in 0.4% sodium chloride immediately prior to use. In placebo-controlled trials, the placebo was 0.9% NaCl, except in two phase II trials, when the placebo was 0.4% NaCl containing 2.5% human serum albumin [9, 15]. The vaccine and placebo were administered by subcutaneous injection in the deltoid region. In trials using a licensed vaccine as an active control, the vaccines were administered according to the usual route of administration.

### Safety assessments

In all trials, after each injection, participants (or parents or legal guardians for children) used diary cards to record the occurrence and severity of solicited injection-site reactions (for 7 days after vaccination), solicited systemic reactions (for 14 days), and unsolicited adverse events (AEs; for 28 days).

Solicited reactions were all considered as being vaccine-related whereas the vaccine-relatedness of unsolicited AEs and SAEs was assessed by the investigators. AEs considered as vaccine-related were called adverse reactions (ARs). AEs occurring within 30 minutes of an injection were considered immediate AEs. Serious adverse events (SAEs), including deaths, and their relatedness were recorded, as specified in each protocol, by investigators.

Solicited injection site reactions included pain, erythema, and swelling, and solicited systemic reactions included fever, headache, myalgia, asthenia, and malaise. In the three efficacy trials, reactogenicity data were collected for participants who had been randomized to the immunogenicity/reactogenicity subset [19–21]. The severity of solicited reactions was graded as 1, 2 or 3 (Table 2). Analyses are reported by age group, defined using the age at enrolment, and, in trials with data available for specific subgroups, analyses are also reported by baseline dengue serostatus and post-injection viremia. Although in the original trials the safety events were coded using different versions of MedDRA, the events were re-coded using version 14.0 to ensure homogeneity across the trials.

**Table 2:** Definitions and severity scales for solicited injection site and systemic reactions.

**Reaction**	**Definition**	**Severity scale**
Injection site pain		**Individuals aged 2–11 years:** *Grade 1*: Easily tolerated; *Grade 2*: Sufficiently discomforting to interfere with normal behavior or activities; *Grade 3*: Incapacitating, unable to perform usual activities. **Individuals aged ≥12 years:** *Grade 1*: No interference with activity; *Grade 2*: Some interference with activity; *Grade 3*: Significant, prevents daily activity
Injection site erythema	Presence of redness including the approximate point of needle entry	**Individuals aged 2–11 years:** *Grade 1*: >0 to <25 mm; *Grade 2*: ≥25 to <50 mm; *Grade 3*: ≥50 mm. **Individuals aged ≥12 years:** *Grade 1*: ≥25 to ≤50 mm; *Grade 2*: ≥51 to ≤100 mm; *Grade 3*: >100 mm
Injection site swelling	Swelling at or near the injection site. Swelling or edema is caused by fluid infiltration in tissue or a cavity and, depending on the space available for the fluid to disperse, swelling may be either soft (typically) or firm (less typical) to the touch and can thus best be described by looking at the size of the swelling	**Individuals aged 2–11 years:** *Grade 1*: >0 to <25 mm; *Grade 2*: ≥25 to <50 mm; *Grade 3*: ≥50 mm. **Individuals aged ≥12 years:** *Grade 1*: ≥25 to ≤50 mm; *Grade 2*: ≥51 to ≤100 mm; *Grade 3*: >100 mm
Fever	Temperature ≥38.0°C	*Grade 1*: ≥38.0°C to ≤38.4°C; *Grade 2*: ≥38.5°C to ≤38.9°C; *Grade 3*: ≥39.0°C
Headache	Pain or discomfort in the head, or scalp. Does not include migraine.	*Grade 1*: No interference with activity; *Grade 2*: Some interference with activity; *Grade 3*: Significant, prevents daily activity
Malaise	General ill feeling of discomfort, illness, or lack of well-being that can be associated with a disease state. It can be accompanied by a sensation of exhaustion or inadequate energy to accomplish usual activities	*Grade 1*: No interference with activity; *Grade 2*: Some interference with activity; *Grade 3*: Significant, prevents daily activity
Myalgia	Muscle aches and pains are common and can involve more than one muscle at the same time. Muscle pain can also involve the soft tissues that surround muscles. These structures, (often referred to as connective tissues) include ligaments, tendons, and fascia. Does not apply to muscle pain at the injection site which should be reported as injection site pain.	*Grade 1*: No interference with activity; *Grade 2*: Some interference with activity; *Grade 3*: Significant, prevents daily activity
Asthenia	Generalized weakness	*Grade 1*: No interference with activity; *Grade 2*: Some interference with activity; *Grade 3*: Significant, prevents daily activity

Allergic reactions and anaphylaxis within 7 days of vaccination and severe dengue disease virologically-confirmed any time after dose 1 were considered as adverse events of special interest (AESIs). Viscerotropic or neurotropic events within 30 days of vaccination (because of the YFV backbone of CYD-TDV) were also considered as AESIs [31, 32]. Episodes of serious dengue disease were defined as acute febrile illness, clinically suspected to be dengue by the investigator before virological confirmation, regardless of severity, but requiring hospitalization (with bed attribution); these events were also recorded as AESIs.

### Biological safety

Biological parameters were assessed at pre-specified time points in subsets of participants in three main trials and all secondary trials (Table 1). Based on changes observed in phase I trials and on biological abnormalities that can mimic dengue disease, the parameters assessed were creatinine, liver function markers, i.e., alanine aminotransferase [ALT] and AST, and bilirubin and hemoglobin [Hb], hematocrit, white blood cells [WBCs], lymphocytes, neutrophils, and platelets. Biological parameters collected from participants with symptomatic dengue disease were not included in this pooled analysis. Since the parameters were assayed by local laboratories using local standards and the normal reference ranges varied between trials, these biological data were standardized for use in the quantitative and toxicity grading analyses. For hematology parameters, the normal ranges in the individual studies were used since there was limited variability across laboratories.

### CYD-TDV vaccine viremia

Vaccine viremia was assessed at pre-specified time points in nine phase I-III trials, in participants with acute febrile illness in six trials conducted in endemic areas (Table 1). Data after doses 1 and 2 were analyzed since vaccine viremia is mainly observed after these injections. Data were analyzed for time points for which vaccine viremia was consistently collected across studies, i.e., D7 (D5-D11) and D14 (D12-D17) in accordance with WHO recommendations [23]. Vaccine viremia was also assessed in individuals with acute febrile episodes within 28 days after vaccination, in trials performed in dengue endemic regions to determine if the fever was vaccine-related (positive vaccine viremia) or was due to dengue infection, in accordance with WHO guidelines [7, 9, 10, 15, 16, 21, 27]. Individuals with positive vaccine viremia, i.e., ≥LLOQ (lower limit of quantitation) measured by YF RT-PCR (non-serotype specific) or CYD RT-PCR (serotype specific) were considered as viremic [16, 19, 21].

### Dengue hospitalization and severe dengue

WHO guidelines stipulate that the clinical evaluation of a candidate live-attenuated dengue tetravalent vaccine should provide evidence that immune response to the vaccine does not predispose vaccinated individuals to develop severe dengue during natural infections in endemic regions [23, 24, 33]. Suspected symptomatic dengue cases were detected using a passive surveillance method in eight phase I/II non-efficacy trials and using an active surveillance system in the phase IIb and two of the phase III efficacy trials [7, 9, 10, 15, 16, 19, 21, 28, 34, 35]. Blood samples were taken from individuals with acute febrile illness (see case definition in Table 1) occurring from 28 days post-dose 1 for virological testing. In the pooled analysis, the relative risk (RR) of virologically-confirmed dengue (hospitalized and/or severe as assessed by the Independent Data Monitoring Committee (IDMC), see next section) occurring up to 25-months post-dose 1 in the phase IIb and two of the phase III efficacy trials was assessed by age group. In addition, virologically-confirmed dengue cases were assessed for severity according to the WHO 1997 recommendations (DHF grades 1–4) [36]. These events occurring in the non-efficacy trials with longer-term follow-up data were summarized using counts and percentages.

In addition, a pooled analysis of the RR of dengue hospitalization and severe dengue among those hospitalized during longer-term follow-up the phase IIb (Years 3 and 4 post-dose 1) and two of the phase III trials (CYD14: Years 3 and 4 post-dose 1; CYD15: Year 3 post-dose 1) by age group at enrolment was performed. The RRs for these endpoints were also calculated for the overall follow-up, i.e. from D0.

### Independent data monitoring committees

WHO guidelines stipulate that an independent data monitoring committee (IDMC) should be set up to ensure the participants’ safety and provide an independent assessment of the safety and efficacy data [23, 24, 33]. Separate IDMCs were set up for each phase I trial whereas a global IDMC was set up for the phase II/IIb and phase III trials to ensure a consistent assessment of the safety profile across all trials in the CYD clinical development program. The IDMC regularly reviewed safety data. Fatal, related SAEs and serious AESIs were reviewed as they occurred. In addition, throughout the CYD-TDV vaccine clinical development program, all dengue cases that were virologically-confirmed were blindly-assessed by the IDMC for disease severity according to pre-defined criteria, as described previously (IDMC severe disease) [19, 20].

### Statistical analysis

No formal testing between groups was performed for reactogenicity and all safety parameters, except severe virologically-confirmed dengue disease, although 95% confidence intervals (CIs) were calculated. The analysis set included all participants with available data who had received ≥1 dose of the dengue vaccine or placebo. Participants were analyzed according to the product received. Sub-group analyzes were performed by age group at enrollment (2–8 years, 9–60 years, 9–17 years and 18–60 years) and for virologically-confirmed dengue from the three efficacy trials, analyses were also performed for those aged 9–16 years. Analyses were also performed by dengue serological status at baseline; individuals with neutralizing antibodies above the low limit of detection (≥10 (1/dil)) against ≥1 dengue serotype at baseline were considered as dengue-seropositive and the others were considered as seronegative.

The statistical methods for estimating the annual incidence rates and relative risk (RR) have been described previously [19–22]. The RR function by age was estimated by kernel smoothing using the univariate Epanechnikov kernel method. Age was considered as a moving window, centered on all the possible ages, a0, and with a size of a0-h and a0+h, where h was 2.0; this approach resulted in a smoothed curve. The weight for each subject was maximal when their age was equal to a0, decreased as the window moved to a0-h and a0+h and was zero outside this window.

## Results

### Characteristics of trials contributing data

In the main clinical trials, 26,356 healthy participants aged between 2 and 60 years at enrollment, received at least one dose of CYD-TDV vaccine and were included in the integrated and pooled analyses (Table 3). The participants in the main trials received 77,234 doses of CYD-TDV and 36,006 doses of placebo. Some participants in the control groups of certain trials received licensed vaccines in accordance with the trial protocols (Tables 1 and 3).

**Table 3:** Number of CYD-TDV (final formulation) injections received overall and by age group in main trials (CYD-TDV at D0 M6 and M12), and all trials (including those who received three doses of CYD-TDV at D0 M3.5/4 and M12) and number of placebo and active control injections.

		CYD-TDV: Main trials	CYD-TDV: All trials	Placebo	Active control*
2–8 years	Total injections	16,816	17,062	7,931	298
	≥1 injection	5,689	5,787	2,772	247
	≥2 injections	5,582	5,673	2,641	51
	3 injections	5,545	5,602	2,518	-
9–17 years	Total injections	56,108	56,403	27,523	433
	≥1 injection	19,120	19,233	9,498	364
	≥2 injections	18,619	18,727	9,196	69
	3 injections	18,369	18,443	8,829	-
18–60 years	Total injections	4,310	5,340	552	535
	≥1 injection	1,547	1,982	335	375
	≥2 injections	1,413	1,758	111	160
	3 injections	1,350	1,600	106	-

* In eight trials some participants in the control group received a licensed vaccine (yellow fever, typhoid, Japanese encephalitis, Tdap, polysaccharide pneumococcal or influenza) as an active control [6, 9, 10, 15, 16, 21, 25–27].

Slightly more participants vaccinated with CYD-TDV were female (51.0%) than male (49.0%), and the mean age was 11.7 years (Table 4). Almost one-third of the participants were Asian (32%) and nearly half were Hispanic with mixed ethnic origins (45%). Among the subset of 7,500 participants tested for dengue sero-status at baseline 59.2% were sero-positive. The demographic characteristics were generally similar between the CYD-TDV and placebo groups, within each age group.

**Table 4:** Demographic and baseline characteristics of participants included in the pooled analysis. Participants in main trials in which the current formulation of the CYD-TDV vaccine was administered at D0; M6 and M12.

		Children (2–8 years)		Adolescents (9–17 years)		Adults (18–60 years)	
Characteristic		CYD-TDV	Placebo	CYD-TDV	Placebo	CYD-TDV	Placebo
	**N**	**5,689**	**2,770**	**19,120**	**9,490**	**1,547**	**302**
Mean age (years)		5.6		11.8	11.7	33.7	31.3
Sex, n (%)	Male	2,748 (48.3)	1,351 (48.8)	9,413 (49.2)	4,643 (48.9)	755 (48.8)	171 (56.6)
	Female	2,941 (51.7)	1,419 (51.2)	9,707 (50.8)	4,847 (51.1)	792 (51.2)	131 (43.4)
Ethnicity^a^, n, %	**N**	**3,689**	**1,804**	**17,776**	**8,857**	**1,006**	**118**
	Asian	3,687 (>99.9)	1,803 (>99.9)	3,360 (18.9)	1,670 (18.9)	144 (14.3)	64 (54.2)
	Black	0.0	0.0	454 (2.6)	226 (2.6)	36 (3.6)	0 (0.0)
	Caucasian	0.0	0.0	1,143 (6.4)	563 (6.4)	782 (77.7)	52 (44.1)
	Hispanic	0.0	0.0	501 (2.8)	249 (2.8)	14 (1.4)	0 (0.0)
	American Indian or Alaska native	0.0	0.0	2,315 (13.0)	1,149 (13.0)	3 (0.3)	0 (0.0)
	Native Hawaiian or Pacific Islander	0.0	0.0	0 (0.0)	0 (0.0)	1 (<0.1)	0 (0.0)
	Other	2 (<0.1)	1 (<0.1)	10,003 (56.3) ^c^	5,000 (56.5)^c^	26 (2.6)	2 (1.7)
Baseline dengue virus status^b^							
Endemic regions	**N**	**1,297**	**591**	**2,842**	**1,369**	**287**	**120**
	Seropositive^d^, n (%)	670 (51.7)	314 (53.1)	2,091 (73.6)	1,021 (74.6)	195 (67.9)	90 (75.0)
	Seronegative, n (%)	627 (48.3)	277 (46.9)	751 (26.4)	348 (25.4)	92 (32.1)	30 (25.0)
Non-endemic regions	**N**	**0**	**0**	**0**	**0**	**877**	**57**
	Seropositive^d^, n (%)	0	0	0	0	71 (8.1)	5 (8.8)
	Seronegative, n (%)	0	0	0	0	806 (91.9)	52 (91.2)

N = number of participants analyzed.

^a^ Ethnicity data were not collected for four studies [9, 10, 14, 15].

^b^ Results from CYD23, CYD14 and CYD15.

^c^ The majority of these individuals were Hispanic, with mixed racial origin mostly driven by the phase III study CYD15 that recruited over 20,000 subjects aged 9 to 16 years

^d^ Presence of neutralizing antibodies any dengue serotype above the lower limit of detection (≥10 (1/dil)) before first injection.

### Solicited injection site and systemic reactions

**Fig 1:**
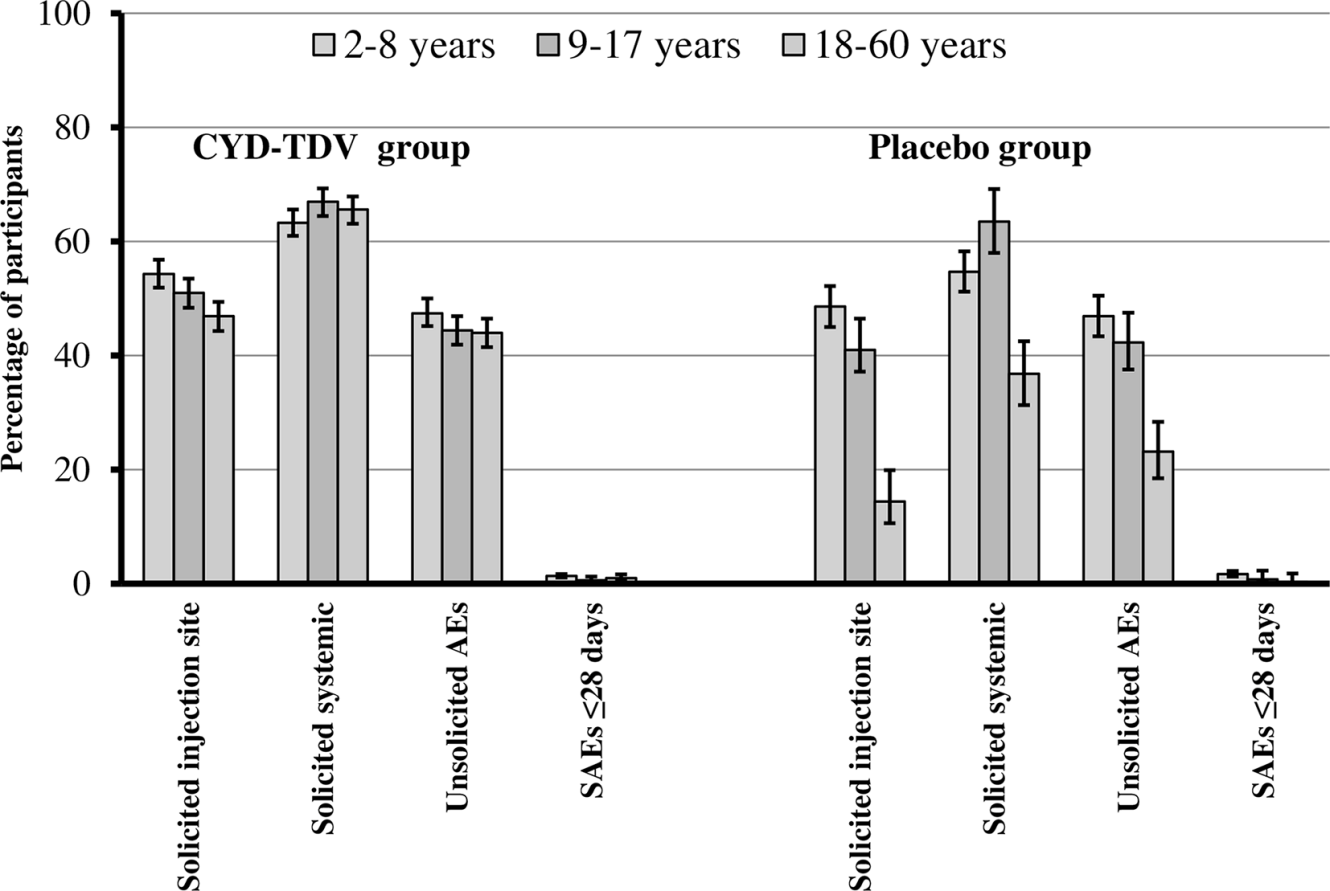
Overall safety profile of CYD-TDV and placebo. Percentages and 95% confidence intervals for participants those who received at least one dose of CYD-TDV (left) or placebo (right) reporting solicited injection-site reactions within 7 days of any dose, solicited systemic reactions within 14 days of any dose, unsolicited AEs and SAEs within 28 days of any dose, by age group.

Overall, in the main trials, there was a trend for a higher percentage of solicited injection-site reactions in participants vaccinated with CYD-TDV (50.9%; 3177/6243) compared with those who received placebo (40.1%; 1018/2537). The highest rates of injection-site reactions were in children in both the CYD-TDV and placebo groups. In both groups, pain was the most common solicited injection-site reaction in all age groups and tended to be more frequent in the CYD-TDV group than in the placebo group (Fig 1; Table 5). Most solicited injection-site reactions were of grade 1 severity, occurred within 3 days of vaccination, and resolved within 3 days.

**Table 5:** Percentage and 95% confidence intervals of participants reporting solicited reactions by age group in the main trials.

		Participants aged (2–8 years)		Participants aged 9–17 years		Participants aged 18–60 years	
Solicited reaction	Severity	CYD-TDV	Placebo	CYD-TDV	Placebo	CYD-TDV	Placebo
Injection-site pain	N	1,669	768	3,050	1,470	1,524	299
	Any	50.1 (47.7; 52.6)	45.6 (42.0; 49.2)	49.2 (47.5; 51.0)	39.0 (36.5; 41.6)	45.2 (42.7; 47.7)	14.4 (10.6; 18.9)
	Grade 3	0.3 (0.1; 0.7)	0.3 (0.0; 0.9)	1.3 (1.0; 1.8)	0.8 (0.4; 1.4)	0.7 (0.4; 1.3	0.3 (0.0; 1.8)
Injection-site erythema	N	1,669	768	3,049	1,470	1,524	299
	Any	19.6 (17.7; 21.6)	18.0 (15.3; 20.9)	8.4 (7.4; 9.4)	7.5 (6.2; 8.9)	7.9 (6.6; 9.3)	0.0 (0.0; 1.2)
	Grade 3	<0.1 (0.0; 0.3)	0.1 (0.0; 0.7)	<0.1 (0.0; 0.2)	<0.1 (0.0; 0.4)	0.0 (0.0; 0.2)	0.0 (0.0; 1.2)
Injection-site swelling	N	1,668	768	3,050	1,470	1,524	299
	Any	14.0 (12.3; 15.7)	12.6 (10.4; 15.2)	6.9 (6.0; 7.8)	5.1 (4.0; 6.4)	2.4 (1.7; 3.3)	0.3 (0.0; 1.8)
	Grade 3	<0.1 (0.0; 0.3)	0.1 (0.0; 0.7)	<0.1 (0.0; 0.3)	<0.1 (0.0; 0.4)	0.0 (0.0; 0.2)	0.0 (0.0; 1.2)
Fever	N	1,667	769	3,040	1,465	1,522	299
	Any	23.0 (21.0; 25.1)	17.7 (15.1; 20.6)	16.4 (15.1; 17.8)	15.6 (13.7; 17.5)	4.9 (3.9; 6.1)	1.3 (0.4; 3.4)
	Grade 3	4.4 (3.5; 5.5)	3.4 (2.2; 4.9)	3.0 (2.4; 3.7)	2.3 (1.6; 3.1)	0.5 (0.2; 1.0)	0.0 (0.0; 1.2)
Headache	N	1,668	768	3,048	1,471	1,524	299
	Any	45.1 (42.7; 47.5)	37.1 (33.7; 40.6)	54.1 (52.3; 55.9)	51.8 (49.2; 54.4)	51.4 (48.9; 54.0)	27.1 (22.1; 32.5)
	Grade 3	1.5 (1.0; 2.2)	1.8 (1.0; 3.0)	6.4 (5.6; 7.3)	4.7 (3.7; 5.9)	6.4 (5.2; 7.7)	2.7 (1.2; 5.2)
Malaise	N	1,668	768	3,047	1,471	1,524	299
	Any	42.6 (40.2; 45.0)	35.7 (32.3; 39.2)	40.9 (39.2; 42.7)	37.5 (35.0; 40.1)	44.3 (41.8; 46.8)	22.1 (17.5; 27.2)
	Grade 3	1.4 (0.9; 2.1)	2.0 (1.1; 3.2)	4.1 (3.4; 4.8)	2.8 (2.0; 3.8)	6.3 (5.1; 7.6)	1.3 (0.4; 3.4)
Myalgia	N	1,668	768	3,047	1,471	1,524	299
	Any	34.7 (32.4; 37.0)	28.9 (25.7; 32.3)	42.0 (40.2; 43.8)	38.1 (35.6; 40.6)	42.2 (39.7; 44.7)	19.4 (15.1; 24.3)
	Grade 3	0.9 (0.5; 1.5)	1.3 (0.6; 2.4)	3.4 (2.8; 4.1)	2.1 (1.4; 3.0)	4.3 (3.4; 5.5)	2.0 (0.7; 4.3)
Asthenia	N	1,668	768	3,047	1,471	1,524	299
	Any	30.3 (28.1; 32.5)	25.7 (22.6; 28.9)	34.2 (32.5; 35.9)	31.3 (28.9; 33.7)	28.3 (26.1; 30.7)	11.7 (8.3; 15.9)
	Grade 3	1.0 (0.6; 1.6)	2.2 (1.3; 3.5)	3.4 (2.8; 4.1)	2.7 (1.9; 3.6)	3.6 (2.7; 4.7)	1.0 (0.2; 2.9)

**Fig 2:**
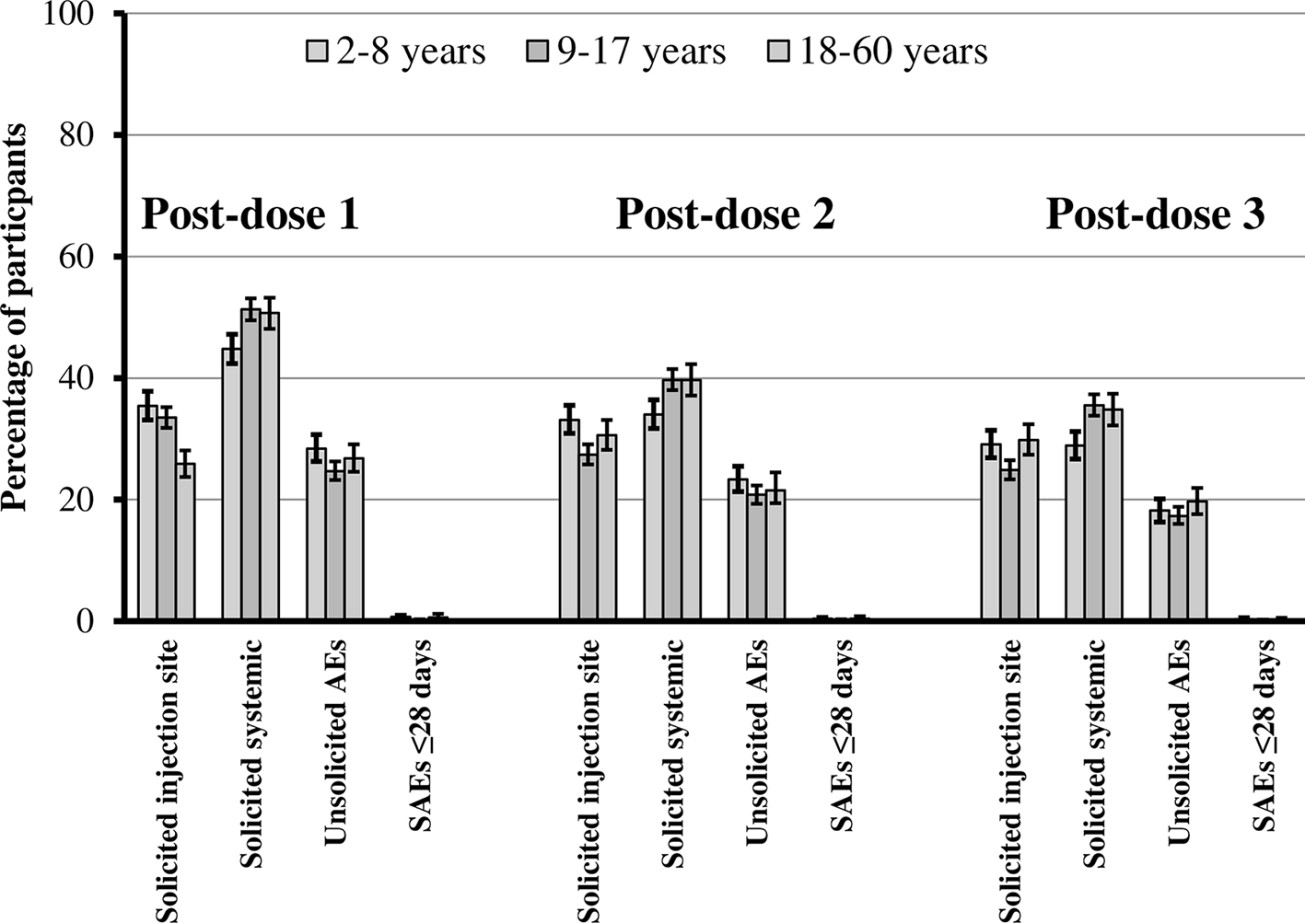
Safety profile after each dose of CYD-TDV. Percentages and 95% confidence intervals for participants those who received at least one dose of CYD-TDV reporting solicited injection-site reactions within 7 days after each dose, solicited systemic reactions within 14 days after each dose, unsolicited AEs and SAEs within 28 days after each dose, by age group children (2–8 years), adolescents (9–17 years) and adults (≥18 years).

There was also a trend for a slightly higher incidence of solicited systemic reactions in the CYD-TDV group (65.7%) compared with the placebo group (57.7%) (Fig 1). Headache and malaise were the most frequently reported solicited systemic reactions in the CYD-TDV and placebo groups (Table 5). The rates of solicited systemic reactions were similar between the age groups except for fever, which was less frequent in adults than in children and adolescents. The highest rate of grade 3 solicited systemic reactions reported for those aged 9–17 years and 18–60 years was headache (6.4%); for children aged 2–8 years it was fever (4.4%). Most solicited systemic reactions occurred within 3 days of injection and resolved within 1–3 days, although fever occurred throughout the solicited period (up to 14 days after vaccination). Overall, the rates of solicited injection site and systemic reactions were higher after the first dose than after the second or third dose (Fig 2).

### Unsolicited adverse events and reactions

Ten (0.3%) and three (0.2%) participants reported immediate unsolicited AEs in the CYD-TDV and placebo groups, respectively; seven (0.1%) and one (<0.1%) participants reported immediate unsolicited ARs, respectively. Unsolicited non-serious AEs were reported for just over 40% of the participants in the CYD-TDV and placebo groups for all age groups, except for those aged 18–60 years in the placebo group (Fig 1). These occurred more frequently after the first dose than after the second or third doses (Fig 2).

Unsolicited non-serious ARs were reported for 4.6% and 1.6% of participants in the CYD-TDV and placebo groups, respectively. The most common were injection-site reactions (hematoma), gastrointestinal disorders, and infections (Table 6). Most of these ARs occurred within 3 days of vaccination, resolved within 3 days, and were grade 1 or 2. The nature of non-serious ARs in the placebo group was similar to that in the CYD-TDV group, but they tended to be less frequent. The rates of non-serious ARs were generally lower after the second and third doses than after the first.

**Table 6:** Unsolicited non-serious adverse reactions affecting ≥0.1% of participants aged 9–60 years in the CYD-TDV and placebo groups. n = number of participants with ≥1 of each SAE.

MedDRA system-organ class	MedDRA preferred term	CYD-TDV group	Placebo group
		N = 4,615	N = 1780
		n, % (95% CI)	n, % (95% CI)
**General disorders and administration site conditions**		**131, 2.8 (2.4; 3.4)**	**15, 0.8 (0.5; 1.4)**
	Injection-site hematoma	51, 1.1 (0.8; 1.5)	7, 0.4 (0.2; 0.8)
	Injection-site pruritus	33, 0.7 (0.5; 1.0)	2, 0.1 (0.0; 0.4)
	Injection-site pain	14, 0.3 (0.2; 0.5)	2, 0.1 (0.0; 0.4)
	Injection-site induration	11, 0.2 (0.1; 0.4)	1, <0.1 (0.0; 0.3)
	Injection-site hemorrhage	4, <0.1 (0.0; 0.2)	2, 0.1 (0.0; 0.4)
**Gastrointestinal disorders**		**30, 0.7 (0.4; 0.9)**	**5, 0.3 (0.1; 0.7)**
	Nausea	12, 0.3 (0.1; 0.5)	2, 0.1 (0.0; 0.4)
	Diarrhea	6, 0.1 (0.0; 0.3)	3, 0.2 (0.0; 0.5)
	Vomiting	6, 0.1 (0.0; 0.3)	2, 0.1 (0.0; 0.4)
**Infections and infestations**		**30, 0.7 (0.4; 0.9)**	**4, 0.2 (0.1; 0.6)**
	Upper respiratory tract infection	9, 0.2 (0.1; 0.4)	1, <0.1 (0.0; 0.3)
	Nasopharyngitis	8, 2 (0.1; 0.3)	1, <0.1 (0.0; 0.3)
**Respiratory, thoracic and mediastinal disorders**		**25, 0.5 (0.4; 0.8)**	**1, <0.1 (0.0; 0.3)**
	Oropharyngeal pain	15, 0.3 (0.2; 0.5)	0, 0.0 (0.0; 0.2)
	Cough	5, 0.1 (0.0; 0.3)	0, 0.0 (0.0; 0.2)
	Rhinorrhea	5, 0.1 (0.0; 0.3)	0, 0.0 (0.0; 0.2)
**Nervous system disorders**		**21, 0.5 (0.3; 0.7)**	**4, 0.2 (0.1; 0.6)**
	Dizziness	10, 0.2 (0.1; 0.4)	1, <0.1 (0.0; 0.5)
**Skin and subcutaneous tissue disorders**		**19, 0.4 (0.2; 0.6)**	**1, <0.1 (0.0; 0.3)**
	Rash	6, 0.1 (0.0; 0.3)	0, 0.0 (0.0; 0.2)
**Musculoskeletal and connective tissue disorders**		**18, 0.4 (0.2; 0.6)**	**1, <0.1 (0.0; 0.3)**
	Arthralgia	5, 0.1 (0.0; 0.3)	0, 0.0 (0.0; 0.2)
	Neck pain	5, 0.1 (0.0; 0.3)	0, 0.0 (0.0; 0.2)
**Blood and lymphatic system disorders**		**7, 0.2 (0.1; 0.3)**	**2, 0.1 (0.0; 0.4)**
	Lymphadenopathy	6, 0.1 (0.0; 0.3)	0, 0.0 (0.0; 0.2)

### Serious adverse events

In the main trials, 218 (0.8%) participants aged 2–60 years in the CYD-TDV group reported ≥1 SAE up to 28-days post any injection and 935 (3.5%) reported ≥1 SAE between day >28 and 6 months post any injection. In the placebo group, there were 121 (1.0%) and 499 (4.0%) participants, respectively. Among participants aged 9–60 years with ≥1 SAE up to 28-days post any injection six and two in the CYD-TDV and placebo groups, respectively (<0.1%) were considered to be vaccine-related SAEs by the investigator; one other SAE (convulsion in a participant aged 9–17 years who had received the CYD-TDV vaccine) was considered to be vaccine-related by the sponsor, but not by the investigator. SAEs considered as vaccine-related by the investigator in the CYD-TDV group were: urticaria, asthma, acute polyneuropathy, tension headache in participants aged 9–17 years; polymyalgia rheumatica and headache in participants aged 18–60 years. In the placebo group, the related SAEs were visual impairment and pyrexia, both in participants aged 9–17 years. In the period between day >28 and 6 months post any injection, one SAE, miscarriage due to blighted ovum considered to be vaccine-related by the investigator occurred in a participant aged 18–60 years in the CYD-TDV group and none in the placebo group. In participants aged 2–8 years, one related SAE was reported in the CYD-TDV group (acute disseminated encephalomyelitis) and two in the placebo group (7th nerve paralysis and angioedema).

The frequency and nature of SAEs occurring within 28 days of any dose were similar in the CYD-TDV and placebo groups. The SAEs were common medical conditions that could be expected as a function of age. The most frequently reported system-organ class was infections and infestations, followed by injuries and gastrointestinal disorders for those aged 9–60 years (Table 7). Among the participants, 22 and 13 had ≥1 neurological SAE within 30-days post-injection in the CYD-TDV and placebo groups, respectively. None of the SAEs resulted in permanent sequelae or death. In addition, a similar profile was observed in those aged 2–8 years.

**Table 7:** SAEs within 28 days after any dose of CYD-TDV vaccine or placebo in participants aged 9–60 years. None of the preferred term events affected ≥0.1% of the participants; n = number of participants with ≥1 of each SAE.

	CYD-TDV	Placebo
	N = 20,667	N = 9,792
MedDRA system-organ class	n, % (95% CI)	n, % (95% CI)
Infections and infestations	64, 0.3 (0.2; 0.4)	29, 0.3 (0.2; 0.4)
Injury, poisoning and procedural complications	21, 0.1 (0.1; 0.2)	11, 0.1 (0.1; 0.2)
Gastrointestinal disorders	16, <0.1 (0.0; 0.1)	3, <0.1 (0.0; 0.1)
Nervous system disorders	16, <0.1 (0.0; 0.1)	9, <0.1 (0.0; 0.2)
Respiratory, thoracic and mediastinal disorders	5, <0.1 (0.0; 0.1)	6, <0.1 (0.0; 0.1)
Psychiatric disorders	4, <0.1 (0.0; 0.1)	4, <0.1 (0.0; 0.1)
Skin and subcutaneous tissue disorders	4, <0.1 (0.0; 0.1)	0, 0.0 (0.0; 0.0)
Immune system disorders	3, <0.1 (0.0; 0.1)	2, <0.1 (0.0; 0.1)
Hepatobiliary disorders	2, <0.1 (0.0; 0.0)	0, 0.0 (0.0; 0.0)
Musculoskeletal and connective tissue disorders	2, <0.1 (0.0; 0.0)	2, <0.1 (0.0; 0.1)
Neoplasms benign, malignant and unspecified (incl cysts and polyps)	2, <0.1 (0.0; 0.0)	1, <0.1 (0.0; 0.1)
Blood and lymphatic system disorders	1, <0.1 (0.0; 0.0)	2, <0.1 (0.0; 0.1)
Congenital, familial and genetic disorders	1, <0.1 (0.0; 0.0)	0, 0.0 (0.0; 0.0)
General disorders and administration site conditions	1, <0.1 (0.0; 0.0)	2, <0.1 (0.0; 0.1)
Pregnancy, puerperium and perinatal conditions	1, <0.1 (0.0; 0.0)	1, <0.1 (0.0; 0.1)
Reproductive system and breast disorders	1, <0.1 (0.0; 0.0)	0, 0.0 (0.0; 0.0)
Eye disorders	0, 0.0 (0.0; 0.0)	1, <0.1 (0.0; 0.1)
Metabolism and nutrition disorders	0, 0.0 (0.0; 0.0)	1, <0.1 (0.0; 0.1)
Renal and urinary disorders	0, 0.0 (0.0; 0.0)	1, <0.1 (0.0; 0.1)

The nature of SAEs observed between day >28 and 6 months post any injection was similar to that observed up to 28 days post any injection. In addition, no safety concern were observed in the review of SAEs during longer-term follow-up, (up to year-3 post-dose 1), particularly in the two phase III efficacy trials, in which all SAEs were recorded.

### Deaths

Six and eight deaths were reported within 6 months after any injection in the CYD-TDV and placebo groups, respectively, in participants aged 2–60 years; none were assessed as related to the CYD-TDV vaccine. In the CYD-TDV group the deaths were due to road traffic accidents (n = 3), tracheal injury (n = 1), deliberate poisoning (n = 1) and accidental asphyxia by strangulation (n = 1). In the placebo group the deaths were due to drowning (n = 2), T-cell lymphoma (n = 1), road traffic accident (n = 1), bronchoscopic aspiration (n = 1), head injury (n = 1), lupus nephritis (n = 1) and metastatic osteosarcoma (n = 1). In the period after 6-months post-dose 3; 17 and 6 deaths occurred in the CYD-TDV and placebo groups, respectively; none were judged to be related to the CYD-TDV vaccine.

### Discontinuations due to SAEs

A total of 71/26,356 (0.3%; 95% CI: 0.21; 0.34) and 33/12,562 (0.3%, 95% CI: 0.18; 0.37) participants in the CYD-TDV and control groups discontinued for safety reasons, including the 14 participants who died (see above). Eight and four participants, respectively, discontinued for SAEs considered related to the vaccine. After the occurrence of the SAE, the individual received no further injections, but continued in the safety surveillance, according to the trial protocol. Trial discontinuations due to vaccine-related SAEs all occurred within 28 days of vaccination, except one which occurred between 7 weeks after vaccination (miscarriage due to blighted ovum) in a participant who had received CYD-TDV.

### Influence of baseline dengue sero-status on safety

**Fig 3:**
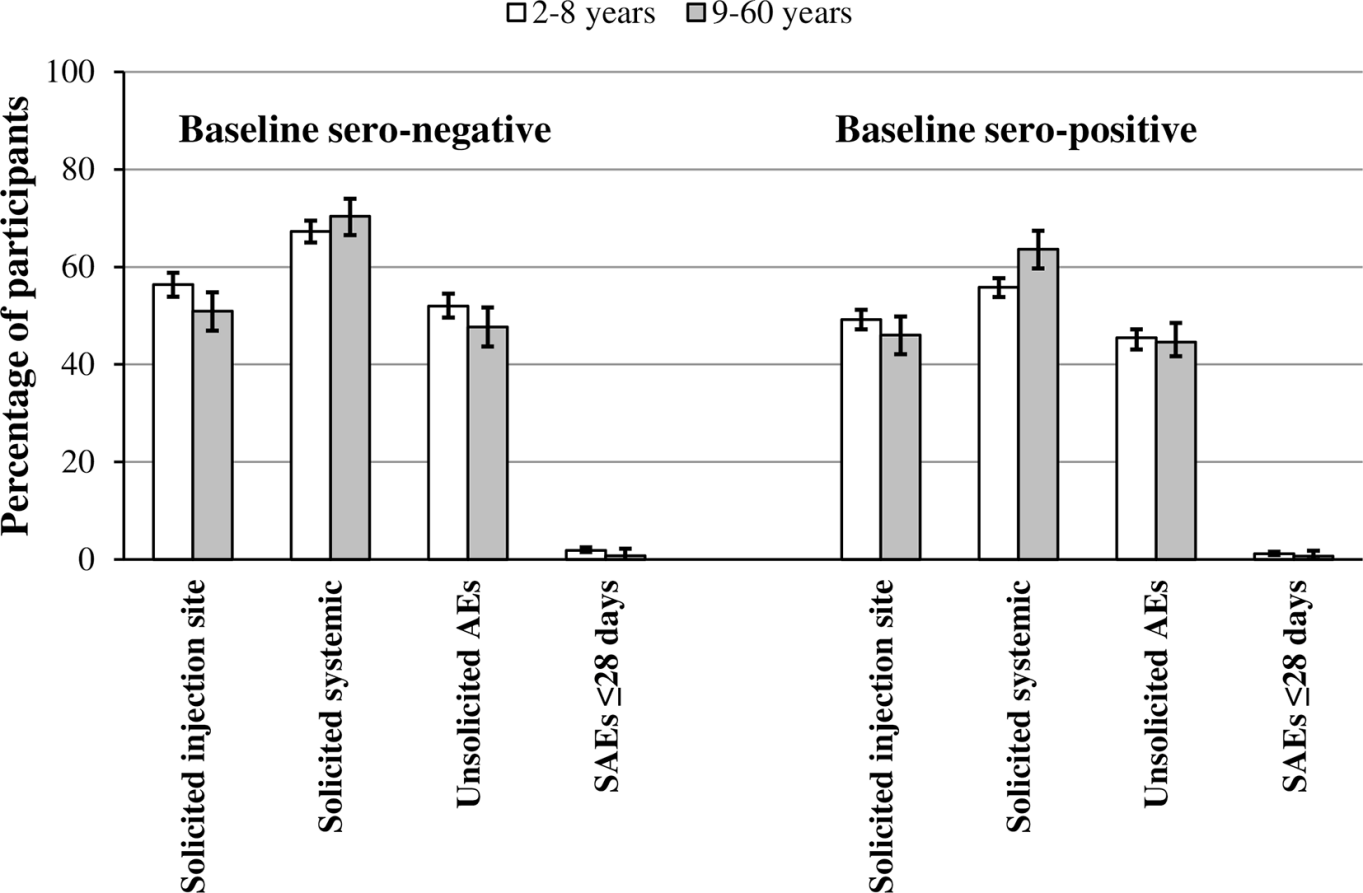
Effect of dengue sero-status at baseline on CYD-TDV safety profile by age. The percentages and 95% confidence intervals for participants who had received at ≥1 dose of CYD-TDV who reported solicited injection-site reactions, solicited systemic reactions, unsolicited non-serious adverse events (AEs) and serious adverse events (SAEs) within 28 days of any dose by age (2–8 years and 9–60 years) and baseline dengue sero-status: sero-negative (left); sero-positive (right)

Baseline dengue virus sero-status did not appear to influence the rates of solicited injection site and systemic reactions, unsolicited non-serious AEs and SAEs in those aged 2–8 and 9–60 years (Fig 3).

### Adverse events of special interest

Among participants aged 9–60 years in the main trials, 46 (0.7%) and 15 (0.5%) experienced non-serious potential allergic reactions (mainly rashes) within 7 days after any injection in the CYD-TDV and placebo groups, respectively. Only 3 were grade 3 (in CYD-TDV group). Most occurred within 3-days post-injection, and resolved spontaneously or after treatment in ≥5 days. In the CYD-TDV group, 14 participants reported these events after dose 1, 5 after dose 2 and 1 after dose 3. Eight of the events in the CYD-TDV group were assessed as vaccine-related; one was grade 3. Five participants (<0.1%) in the CYD-TDV group experienced serious allergic reactions (four experienced asthma or asthmatic crisis and all had a medical history of asthma, asthmatic bronchitis, or bronchial obstructive symptoms; one experienced urticaria and had a history of allergic rhinitis). One participant in the placebo group experienced asthma. A similar profile for AESIs was observed in participants aged 2–8 years. All participants recovered spontaneously or after medical care. Overall, no severe or serious immediate anaphylactic reactions following CYD-TDV vaccination were reported in any age group.

No confirmed cases of viscerotropic or neurotropic disease were reported.

The reporting of serious dengue disease, occurring at any time during the trials, as an AESI was implemented in the four phase III trials (Table 1). In two of these trials (non-efficacy), CYD17 and CYD32, that enrolled 250 and 715 participants, respectively, no serious dengue disease was reported over the 18-month follow-up period. In CYD14, 38/50 (76%) and 56/64 (88%) of serious dengue disease events in the CYD-TDV and placebo groups, respectively, that occurred up to 25-months post-dose 1 were virologically-confirmed. In CYD15, 14/41 (34%) and 38/50 (76%) serious dengue events, respectively, were virologically-confirmed.

### Biological safety profile

The pooled analyses of biological data available for 676 participants showed that most values were within normal ranges both at baseline and after any CYD-TDV dose. The highest rates of grade 3 biological abnormal values reported were 2.2% for low hemoglobin (15/668) and 1.8% for neutrophils (12/668), with no specific patterns being observed. The abnormal values for 12 participants were assessed as being vaccine-related, but none were reported as an SAE within 28 days after any CYD-TDV dose.

In addition, in individual trials, the biological safety profile of the CYD-TDV vaccine was found to be similar to that of the control groups (placebo or licensed vaccines). The incidence of biological abnormalities was 73.7% in participants with viremia compared with 74.8% in those without viremia.

### Vaccine viraemia

The pooled analyses of viremia data available for 683 participants showed that 38 subjects (<6.0%) had detectable vaccine viremia after dose 1 or 2 of the CYD–TDV vaccine (34 after dose 1; 4 after dose 2). All levels of vaccine viremia were low. None of the participants with viremia experienced immediate AEs, post-vaccination dengue-like syndrome, AEs leading to trial discontinuation, AESIs, or SAEs. The rates of solicited reactions, non-serious unsolicited AEs, non-serious unsolicited ARs were similar between viremic and non-viremic participants (63.2% vs. 69.3%; 63.2% vs. 63.9%; 13.2% vs. 12.1%, respectively). Overall, no safety concerns were identified in the participants with vaccine viremia.

In six trials, blood samples were collected from participants who experienced an acute febrile episode (as defined in each protocol) within 28 days following dose 1 (n = 113) or 2 (n = 106), to be tested for wild-type and vaccine dengue viremia [7, 9, 10, 15, 16, 21, 27]. Vaccine viremia was detected in only one participant (after dose 1). This participant did not have virologically-confirmed dengue disease and had no reports of safety outcomes.

### Dengue hospitalization and severe dengue up to 25-months post-dose 1

In the phase IIb and phase III efficacy trials, 89 and 134 individuals in the CYD-TDV and placebo groups, respectively, were hospitalized during the 25-month period after dose 1, with a RR of 0.33 (95% CI: 0.25, 0.43) in vaccine recipients. In these trials, there were 15 and 33 cases of severe dengue disease (IDMC assessment) in the CYD-TDV and placebo groups, respectively, with a RR of 0.23 (95% CI: 0.12; 0.42). In the CYD-TDV and placebo groups, 11 and 10 severe dengue cases, respectively, were reported in those aged 2–8 years; in those aged 9–16 there were 4 and 23 cases, respectively. There was no evidence of increased severity of dengue disease based on the review of the severity of clinical outcomes, biological parameters, vaccine viremia and hospitalization rates [19–21].

In the other phase I/II/III non-efficacy trials with a passive surveillance, very few hospitalized virologically-confirmed dengue cases were reported in the CYD-TDV group up to 6 month post-dose 3. None was assessed as severe by IDMC.

### Dengue hospitalization and severe dengue during longer-term follow-up

The planned longer-term follow-up for participants in the efficacy trials is on-going. In the follow-up study for the phase IIb trial (CYD23/57), and in the phase III study in Asia (CYD14) data for virologically-confirmed dengue disease hospitalization and severe dengue disease hospitalization are available for two-years of longer-term follow-up (i.e. four-years after dose 1). In the phase III trial in Latin America (CYD15), data are available for one-year of longer-term follow-up (i.e. three-years after dose 1). The participants were originally randomized 2:1 to receive CYD-TDV or placebo.

#### Dengue hospitalization and severe dengue during Year 3

During year 3 of these trials, among all participants (aged 2–16 years), the overall pooled RR for hospitalization for symptomatic, virologically-confirmed dengue was 0.84 (95% CI: 0.56; 1.24) with 65/22,177 (0.29%) in the vaccine group and 39/11,089 (0.35%) in the control group [22]. Among those aged <9 years, the pooled RR was 1.58, 95% CI: 0.83; 3.02, and among those aged ≥9 years the RR was 0.50, 95% CI: 0.29; 0.86. Overall, during year 3, hospitalization for severe dengue occurred in 18/22,177 (0.08%) and 6/11,089 (0.05%) participants in the CYD-TDV and control groups, respectively. All participants with severe dengue disease recovered after appropriate treatment.

In the Asian phase III trial, the IDMC classified 12 cases of hospitalized dengue disease, in year 3, as severe (CYD-TDV, n = 11; placebo, n = 1; RR = 5.50 [95% CI: 0.80; 236.60]). Four of the 12 severe cases occurred in individuals aged 9–14 years (CYD-TDV, n = 3; placebo, n = 1) and the remaining eight occurred in the CYD-TDV group in those aged 2–8 years showing an excess of severe hospitalized cases in the CYD-TDV group. These cases were all classified as WHO DHF grade 1 (n = 5) or 2 (n = 7). This includes one of the participants in the CYD-TDV group, aged 12 years at enrollment, who presented with clinical shock which was classified as DHF grade 2.

In the Latin American phase III trial, which enrolled participants aged 9–16 years, the IDMC classified eight cases of hospitalized dengue disease, as severe in year 3 (3 and 5 in the CYD-TDV and placebo groups, respectively) with a RR of 0.30 (95% CI: 0.05; 1.54). These cases were all classified as DHF grade 2.

In the phase IIb trial, the IDMC classified four cases of hospitalized dengue disease in year 3 as severe, all in participants aged 4–8 years in the CYD-TDV group. One case was classified DHF grade 1, one DHF grade 2 and the remaining two of these cases were classified as DHF grade 3 associated with clinical shock.

#### Dengue hospitalization and severe dengue during Year 4

In year 4 of the phase IIb study, 16 and 17 participants in the CYD-TDV and placebo groups, respectively, were hospitalized for dengue (RR = 0.47, 95% CI: 0.22; 1.00). The RR for participants aged <9 years was 0.54 (95% CI: 0.23; 1.29) and 0.31 (95% CI: 0.05; 1.58) for those aged ≥9 years. The IDMC classified dengue disease as severe in three of these hospitalized participants (CYD-TDV, n = 1; placebo, n = 2); all occurred in patients aged <9 years at enrollment (RR = 0.25, 95% CI: 0.00; 4.83) and were classified as DHF grade 1 or 2.

In year 4 of the phase III trial, CYD14, 57 and 29 participants in the CYD-TDV and placebo groups, respectively, were hospitalized for dengue (RR = 0.98, 95% CI: 0.62; 1.59). The RR for participants aged <9 years was 1.19 (95% CI: 0.65; 2.28) and 0.73 (95% CI: 0.34; 1.61) for those aged ≥9 years. The IDMC classified 19 of these cases in the hospitalized participants as severe (CYD-TDV, n = 13; placebo, n = 6) RR = 1.08 (0.38; 3.47); 13 cases occurred in patients aged <9 years at enrollment (CYD-TDV, n = 9; placebo, n = 4) and 6 occurred in patients aged ≥9 years at enrollment (CYD-TDV, n = 4; placebo, n = 2). One case was not classified as DHF (associated with clinical shock; CYD-TDV group; aged<9 years at enrollment) and the others were all classified as DHF grade 1 (n = 3) and grade 2 (n = 15) including two associated with clinical shock (both in the placebo group, one aged <9 years and one aged ≥9 years at enrollment).

#### Dengue hospitalization and severe dengue from Dose 1 to Year 4

For the entire period from Dose 1 to Year 4, the cumulative RR for hospitalization was 0.61 (95% CI: 0.42; 0.87) and 0.60 (95% CI: 0.46; 0.79) for the phase IIb and the Asian phase III trials, respectively. In these trials the RRs for those aged <9 years were 0.78 (95% CI: 0.51; 1.21) and 0.79 (95% CI: 0.56; 1.13), respectively. In the Latin American phase III trial from Dose 1 to Year 3, the cumulative RR for hospitalization was 0.28 (95% CI: 0.18; 0.44). The overall RRs for participants aged ≥9 years were similar in all three trials, with RR <0.4 and the upper bound of the 95% CI below 1.0.

### Dengue hospitalization and severe dengue in the other trials

Only one hospitalized virologically-confirmed dengue case, assessed as non-severe by IDMC, was reported in the CYD-TDV group, during the longer-term follow-up (from 6 months after the last injection up to year 5) in one of the three non-efficacy phase I-II trials.

### Cumulative dengue hospitalization (any severity) and severe dengue

**Fig 4:**
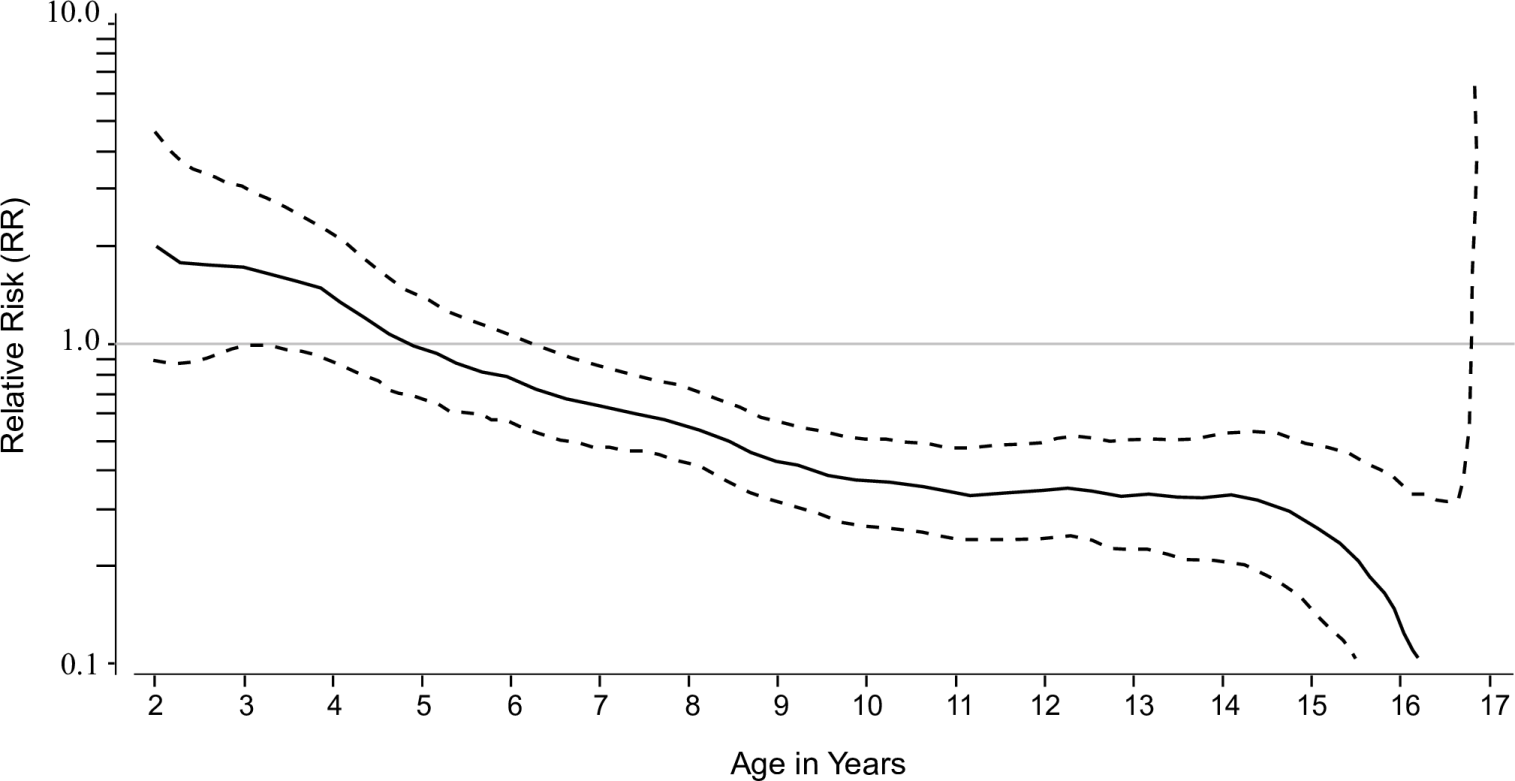
Relative risk (solid line) and 95% confidence interval (dotted lines) against hospitalized or severe dengue due to any serotype by age at enrollment. The Epanechnikov kernel method (with h = 2.0) was used to provide a smooth curve. Data were combined from post-dose 1 up to year 3 in the phase III trial in Latin America and up to year 4 in the phase IIb and phase III trial in Asia.

The cumulative RR for hospitalization for dengue disease by age at enrollment was analyzed from D0 up to year 3 in the phase III trial in Latin America and year 4 in the phase IIb and phase III trials in Asia (Fig 4). The RR for dengue hospitalization or severe dengue disease by age at enrollment decreased to below 1 at about age 5 and the 95% CI was below 1 from age 6 onwards, demonstrating an overall reduction in risk of dengue hospitalization and severe dengue in those aged ≥6 years (Fig 4).

## Discussion

This pooled analyses included data from 13 main and 5 secondary clinical trials with 77,234 doses of the current formulation of the CYD-TDV vaccine (about 5 log_10_ CCID_50_ of each of the four live, attenuated dengue vaccine viruses) in individuals aged between 2 and 60 years. The results showed that the overall reactogenicity and safety profile for the CYD-TDV vaccine, including solicited reactions, unsolicited and serious adverse events, viremia and biological parameters was satisfactory and comparable to that for placebo across all age groups (2–8 years; 9–60 years) with similar reported rates and nature of events in the CYD-TDV and placebo groups. The safety profile was not influenced by baseline dengue sero-status or successive doses of the CYD-TDV vaccine. In addition, the reported AEs were transient and mainly mild to moderate. No safety concerns were identified for the frequency and nature of unsolicited AEs and SAEs. The nature of the reported SAEs was consistent with the participants’ ages, with few being considered as vaccine-related. There were no vaccine-related deaths. Likewise, no severe or serious immediate anaphylactic reactions to CYD-TDV were reported, although rash, an event that may indicate an allergic reaction, was reported by a similar percentage of participants in both groups.

Viscerotropic and neurotropic diseases are very rare events that have been reported for YFV vaccines such as Stamaril [37]. The event rate after vaccination with a licensed YFV vaccine has been reported to be 0.4/100,000 doses for viscerotropic events and 0.8/100,000 doses for neurotropic events [38]. Since the CYD-TDV vaccine viruses cannot express the YFV E protein, which is largely responsible for YFV tropism, it is unlikely that they will display the same tropism as YFV and none were reported in any age group [39]. Nevertheless, monitoring for these very rare events will continue after vaccine introduction through post-marketing surveillance in real life settings.

Vaccine viremia due to vaccination with live attenuated tetravalent dengue vaccines is considered by the WHO to be unlikely to cause dengue disease due to poor replication of the attenuated viruses [24]. The results presented here show that vaccine viremia was observed in <6% of the participants with available data, and the safety profiles were similar between participants with viremia and those without viremia. In addition, no cases of virologically-confirmed dengue disease were reported among the participants with viremia.

Several risk factors for severe dengue following natural exposure to dengue infection have been identified, including previous infection with a different serotype [24]. Approximately 2% to 4% of patients who have a secondary infection with a heterologous type of dengue virus develop more severe illness [40]. Several hypotheses have been put forward to explain this phenomenon including antibody-dependent enhancement (ADE) [1, 41, 42]. ADE has been observed in mouse models and monkeys, but only indirect evidence is available for this phenomenon in humans [43–46]. As specified by the WHO guidelines, we assessed if the immune response to the live attenuated tetravalent dengue vaccines predisposed individuals in endemic regions to more severe dengue disease [23, 33]. In this regard, a reduction in the rates of dengue hospitalization or severe dengue was observed in the 25-month period post-dose 1 in participants in the CYD-TDV group compared with those in the placebo group. This reduction in rates of dengue hospitalization and severe dengue has continued up to Years 3 and 4 post-dose 1 in vaccinated children aged ≥9. In contrast, the data up to Year 3 from the phase IIb and phase III trials in Asia showed that there was an imbalance of hospitalization and severe dengue in younger children aged <9 years, mainly driven by participants aged <6 years [22]. However, data from Year 4 in these two trials, that enrolled participants aged <9 years, no longer show this imbalance for the younger participants, with the RR for hospitalization decreasing from 1.57 in Year 3 to 0.54 in the phase IIb trial and from 1.58 to 1.19 in the phase III trial. In addition, there were 12 vs. 0 participants with severe dengue in the CYD-TDV and placebo groups, respectively in Year 3, compared with 10 vs. 6 in Year 4. Within the setting of the clinical trials we have closely monitored safety during pre-defined periods (i.e. yearly). However, it is important to look at the overall safety profile in terms of value of the vaccine which shows that rates for hospitalization and severe dengue from post-dose 1 to the end of Year 4 were lower in the CYD-TDV group compared with the placebo group, overall and by age group (<9 years and ≥9 years).

Some interconnected mechanisms, involving interactions between the infecting virus, pre-existing host immunity and vaccine-induced immune responses, have been proposed to explain the Year 3 observations in participants aged <9 years. [47, 48]. Although there is no conclusive evidence yet to support a particular mechanism for this phenomenon, our observations from the Year 4 data showing a decreased RR would seem to support the hypothesis that clustered vaccination in young vaccines, which may act as a primary-like exposure, would result in an ‘accelerated secondary infection’ in that group compared with the placebo group. In the placebo group, the ‘accelerated secondary infection’ would eventually occur at a later time point, making the observed Year 3 imbalance only temporary [48]. It is essential to note that there were no important differences in the clinical pattern and outcomes of severity (e.g. bleeding, thrombocytopenia, shock, plasma leakage, duration of symptoms, duration of hospitalization), biological parameters and presence of viremia for the cases of dengue hospitalization and severe dengue in all participants irrespective of the age, group and observation period; all subjects with severe dengue fully recovered [22]. In addition, the measurement of 38 cytokines, chemokines and growth factors did not reveal any particular immune risk profile in those who had received CYD-TDV vaccine [49]. We observed no differences in the profiles of these factors measured in acute sera from vaccine and placebo recipients who had been hospitalized for dengue or who had severe dengue, irrespective of trial, observation period, severity and age, which is consistent with the clinical findings and viremia results.

The cumulative RR by age showed that there was an overall reduction in risk of dengue hospitalization and severe dengue in those aged ≥6 years. In a conservative approach, a safety margin has been integrated in the vaccine’s indication which is for subjects aged 9 years or more. At the time of manuscript submission, these safety data have supported the licensure of this vaccine, Dengvaxia, in individuals aged 9 to 45 years in Mexico, the Philippines, Brazil and El Salvador and in individuals aged 9 to 60 years in Paraguay, making it the first vaccine to be licensed for the prevention of disease caused by four dengue virus serotypes. The WHO Strategic Advisory Group of Experts (SAGE) on Immunization reviewed the data for CYD-TDV in April 2016 and recommended countries consider introduction of the vaccine in geographic settings (national or subnational) with high endemicity [50]. A WHO vaccine position paper will be published outlining their recommendations in July 2016. In accordance with WHO recommendations, safety and efficacy follow-up will continue for five years after the third dose in the phase IIb efficacy trial and the two phase III efficacy trials in the context of the post-licensure surveillance [23, 33].

To evaluate the safety and effectiveness including indirect effects of the vaccine in ‘real-life’ setting, the post-licensure plan, summarized in the pharmacovigilance risk management plan includes post-authorization safety and effectiveness studies, which will be planned in close collaboration with national health authorities, in addition to routine pharmacovigilance surveillance. This surveillance, which will be implemented once the vaccine has been introduced, will provide data for larger-scale reactogenicity and safety assessments of the CYD-TDV vaccine in real-world settings, including in populations excluded from clinical trials. The pooled safety database was large enough to allow detection of uncommon adverse events occurring in at least 1 per 1,000 individual. As longer-term post-marketing safety data become available the detection of any rare and unexpected events will be also possible.

The results from this integrated analysis show that the CYD-TDV vaccine has satisfactory, short- and long-term reactogenicity and safety profiles for up to four years post-dose 1 in participants aged 9–60 years.

## Supporting Information

S1 Table Summary safety data from CYD51 trial (NCT01488890): phase II, randomized, open-label, multicenter trial with active control. (DOCX)
