# Supplementary material for: Safety Overview of a Recombinant Live-Attenuated Tetravalent Dengue Vaccine: Pooled Analysis of Data from 18 Clinical Trials
Source: PLoS Negl Trop Dis. 2016 Jul 14;10(7):e0004821. doi: 10.1371/journal.pntd.0004821 (PMC4945086; doi:10.1371/journal.pntd.0004821)
Supplement: S1 Table — (DOCX) [file pntd.0004821.s001.docx]

**Summary safety data from CYD51 trial (NCT01488890): phase II, randomized, open-label, multicenter trial with active control**

**Table S1:** Safety overview in the CYD group (3 doses at M0, M6 and M12) and in active control group after yellow fever (YF) dose at M0

| **Participants experiencing at least one:** | **CYD group**  **1st dose M0**  **N=120** | |  | **YF group**  **1 dose M0**  **N=30** | |  | **CYD group**  **2nd dose M6**  **N=105** | |  | **CYD group**  **3rd dose M12**  **N=98** | |
| --- | --- | --- | --- | --- | --- | --- | --- | --- | --- | --- | --- |
|  | **n/M** | **% (95% CI)** |  | **n/M** | **% (95% CI)** |  | **n/M** | **% (95% CI)** |  | **n/M** | **% (95% CI)** |
| **Immediate unsolicited AE** | 0/120 | 0.0 (0.0; 3.0) |  | 0/30 | 0.0 (0.0; 11.6) |  | 0/105 | 0.0 (0.0; 3.5) |  | 0.0 | 0.0 (0.0; 3.7) |
| **Immediate unsolicited AR** | 0/120 | 0.0 (0.0; 3.0) |  | 0/30 | 0.0 (0.0; 11.6) |  | 0/105 | 0.0 (0.0; 3.5) |  | 0.0 | 0.0 (0.0; 3.7) |
| **Solicited reaction** | 75/118 | 63.6 (54.2; 72.2) |  | 16/27 | 59.3 (38.8; 77.6) |  | 44/101 | 43.6 (33.7; 53.8) |  | 39.6 | 39.6 (29.5; 50.4) |
| **Solicited injection site reaction** | 34/118 | 28.8 (20.8; 37.9) |  | 10/27 | 37.0 (19.4; 57.6) |  | 21/101 | 20.8 (13.4; 30.0) |  | 20.9 | 20.9 (13.1; 30.7) |
| **Solicited systemic reaction** | 65/118 | 55.1 (45.7; 64.3) |  | 16/27 | 59.3 (38.8; 77.6) |  | 38/101 | 37.6 (28.2; 47.8) |  | 35.2 | 35.2 (25.4; 45.9) |
| **Unsolicited AE *** | 34/120 | 28.3 (20.5; 37.3) |  | 10/30 | 33.3 (17.3; 52.8) |  | 15/105 | 14.3 (8.2; 22.5) |  | 19.4 | 19.4 (12.1; 28.6) |
| **Unsolicited AR** | 7/120 | 5.8 (2.4; 11.6) |  | 3/30 | 10.0 (2.1; 26.5) |  | 3/105 | 2.9 (0.6; 8.1) |  | 2.0 | 2.0 (0.2; 7.2) |
| **Unsolicited non-serious AE *** | 34/120 | 28.3 (20.5; 37.3) |  | 10/30 | 33.3 (17.3; 52.8) |  | 15/105 | 14.3 (8.2; 22.5) |  | 19.4 | 19.4 (12.1; 28.6) |
| **Unsolicited non-serious AR** | 7/120 | 5.8 (2.4; 11.6) |  | 3/30 | 10.0 (2.1; 26.5) |  | 3/105 | 2.9 (0.6; 8.1) |  | 2.0 | 2.0 (0.2; 7.2) |
| **Unsolicited non-serious injection site AR** | 4/120 | 3.3 (0.9; 8.3) |  | 2/30 | 6.7 (0.8; 22.1) |  | 1/105 | 1.0 (0.0; 5.2) |  | 2.0 | 2.0 (0.2; 7.2) |
| **Unsolicited non-serious systemic AE *** | 31/120 | 25.8 (18.3; 34.6) |  | 10/30 | 33.3 (17.3; 52.8) |  | 15/105 | 14.3 (8.2; 22.5) |  | 0.0 | 0.0 (0.0; 3.7) |
| **Unsolicited non-serious systemic AR** | 3/120 | 2.5 (0.5; 7.1) |  | 1/30 | 3.3 (0.1; 17.2) |  | 2/105 | 1.9 (0.2; 6.7) |  | 17.3 | 17.3 (10.4; 26.3) |
| **AE leading to study discontinuation †** | 0/120 | 0.0 (0.0; 3.0) |  | 0/30 | 0.0 (0.0; 11.6) |  | 0/105 | 0.0 (0.0; 3.5) |  | 0.0 | 0.0 (0.0; 3.7) |
| **SAE ‡** | 1/120 | 0.8 (0.0; 4.6) |  | 0/30 | 0.0 (0.0; 11.6) |  | 0/105 | 0.0 (0.0; 3.5) |  | 0.0 | 0.0 (0.0; 3.7) |
| **Death** | 0/120 | 0.0 (0.0; 3.0) |  | 0/30 | 0.0 (0.0; 11.6) |  | 0/105 | 0.0 (0.0; 3.5) |  | 0.0 | 0.0 (0.0; 3.7) |
| **AESI §** | 0/120 | 0.0 (0.0; 3.0) |  | 0/30 | 0.0 (0.0; 11.6) |  | 0/105 | 0.0 (0.0; 3.5) |  | 0.0 | 0.0 (0.0; 3.7) |
| **Non-serious AESI** | 0/120 | 0.0 (0.0; 3.0) |  | 0/30 | 0.0 (0.0; 11.6) |  | 0/105 | 0.0 (0.0; 3.5) |  | 0.0 | 0.0 (0.0; 3.7) |
| **Serious AESI** | 0/120 | 0.0 (0.0; 3.0) |  | 0/30 | 0.0 (0.0; 11.6) |  | 0/105 | 0.0 (0.0; 3.5) |  | 0.0 | 0.0 (0.0; 3.7) |

n: number of subjects experiencing the endpoint listed in the first column

M: number of subjects with available data for the relevant endpoint

AE: adverse events; AR: adverse reactions; SAE: serious adverse event; AESI: adverse event of special interest

*AE collected within 28 days after the first injection

† Identified in the termination form as SAE or other AE up to 28 days after the first injection

‡ Includes SAEs and serious AESIs collected up to 28 days after the first injection

§ Includes non-serious and serious AESIs collected up to 28 days after the first injection

**Table S2:** All serious adverse events and serious adverse events of interest during the trial after any dose by system organ class and preferred term

| Participants experiencing at least one: | **CYD group**  **N=120** | | |  | **YF group**  **N=30** | | |
| --- | --- | --- | --- | --- | --- | --- | --- |
|  | **n** | **% (95% CI)** | **n SAEs** |  | **n** | **% (95% CI)** | **n SAEs** |
| **Serious adverse event** | **4** | **3.3 (0.9; 8.3)** | **6** |  | **0** | **0.0 (0.0; 11.6)** | **0** |
| Hepatobiliary disorders | 1 | 0.8 (0.0; 4.6) | 1 |  | 0 | 0.0 (0.0; 11.6) | 0 |
| *Cholecystitis acute* | 1 | 0.8 (0.0; 4.6) | 1 |  | 0 | 0.0 (0.0; 11.6) | 0 |
| Infections and infestations | 2 | 1.7 (0.2; 5.9) | 3 |  | 0 | 0.0 (0.0; 11.6) | 0 |
| *Abdominal abscess* | 0 | 0.0 (0.0; 3.0) | 0 |  | 0 | 0.0 (0.0; 11.6) | 0 |
| *Appendicitis* | 1 | 0.8 (0.0; 4.6) | 1 |  | 0 | 0.0 (0.0; 11.6) | 0 |
| *Diverticulitis* | 1 | 0.8 (0.0; 4.6) | 1 |  | 0 | 0.0 (0.0; 11.6) | 0 |
| *Pneumonia* | 1 | 0.8 (0.0; 4.6) | 1 |  | 0 | 0.0 (0.0; 11.6) | 0 |
| Injury, poisoning and procedural complications | 0 | 0.0 (0.0; 3.0) | 0 |  | 0 | 0.0 (0.0; 11.6) | 0 |
| *Femur fracture* | 0 | 0.0 (0.0; 3.0) | 0 |  | 0 | 0.0 (0.0; 11.6) | 0 |
| *Rib fracture* | 0 | 0.0 (0.0; 3.0) | 0 |  | 0 | 0.0 (0.0; 11.6) | 0 |
| *Toxicity to various agents* | 0 | 0.0 (0.0; 3.0) | 0 |  | 0 | 0.0 (0.0; 11.6) | 0 |
| Neoplasms benign, malignant and unspecified (incl cysts and polyps) | 1 | 0.8 (0.0; 4.6) | 1 |  | 0 | 0.0 (0.0; 11.6) | 0 |
| *Basal cell carcinoma* | 0 | 0.0 (0.0; 3.0) | 0 |  | 0 | 0.0 (0.0; 11.6) | 0 |
| *Breast cancer* | 0 | 0.0 (0.0; 3.0) | 0 |  | 0 | 0.0 (0.0; 11.6) | 0 |
| *Squamous cell carcinoma* | 0 | 0.0 (0.0; 3.0) | 0 |  | 0 | 0.0 (0.0; 11.6) | 0 |
| *Vaginal cancer* | 1 | 0.8 (0.0; 4.6) | 1 |  | 0 | 0.0 (0.0; 11.6) | 0 |
| Pregnancy, puerperium and perinatal conditions | 1 | 0.8 (0.0; 4.6) | 1 |  | 0 | 0.0 (0.0; 11.6) | 0 |
| *Blighted ovum* | 1 | 0.8 (0.0; 4.6) | 1 |  | 0 | 0.0 (0.0; 11.6) | 0 |
| Psychiatric disorders | 0 | 0.0 (0.0; 3.0) | 0 |  | 0 | 0.0 (0.0; 11.6) | 0 |
| *Suicidal ideation* | 0 | 0.0 (0.0; 3.0) | 0 |  | 0 | 0.0 (0.0; 11.6) | 0 |
| Respiratory, thoracic and mediastinal disorders | 0 | 0.0 (0.0; 3.0) | 0 |  | 0 | 0.0 (0.0; 11.6) | 0 |
| *Asthma* | 0 | 0.0 (0.0; 3.0) | 0 |  | 0 | 0.0 (0.0; 11.6) | 0 |
| *Atelectasis* | 0 | 0.0 (0.0; 3.0) | 0 |  | 0 | 0.0 (0.0; 11.6) | 0 |
| *Pneumothorax* | 0 | 0.0 (0.0; 3.0) | 0 |  | 0 | 0.0 (0.0; 11.6) | 0 |
| Vascular disorders | 0 | 0.0 (0.0; 3.0) | 0 |  | 0 | 0.0 (0.0; 11.6) | 0 |
| *Thrombosis* | 0 | 0.0 (0.0; 3.0) | 0 |  | 0 | 0.0 (0.0; 11.6) | 0 |

n: number of subjects experiencing the event; n SAEs: number of SAEs
